# Immunoconjugates for Osteosarcoma Therapy: Preclinical Experiences and Future Perspectives

**DOI:** 10.3390/biomedicines6010019

**Published:** 2018-02-10

**Authors:** Daniele Mercatelli, Massimo Bortolotti, Alberto Bazzocchi, Andrea Bolognesi, Letizia Polito

**Affiliations:** 1 Department of Experimental, Diagnostic and Specialty Medicine-DIMES, Alma Mater Studiorum, University of Bologna, Via San Giacomo 14, 40126 Bologna, Italy; daniele.mercatelli@ior.it (D.M.); massimo.bortolotti2@unibo.it (M.B.); andrea.bolognesi@unibo.it (A.B.); 2 Department of Diagnostic and Interventional Radiology, The “Rizzoli” Orthopaedic Institute, Via G. C. Pupilli 1, 40136 Bologna, Italy; abazzo@inwind.it

**Keywords:** osteosarcoma, immunoconjugates, immunotherapy, cancer therapy

## Abstract

Osteosarcoma (OS) is an aggressive osteoid-producing tumor of mesenchymal origin, which represents the most common primary bone malignancy. It is characterized by a complex and frequently uncertain etiology. The current standard care for high-grade OS treatment is neoadjuvant chemotherapy, followed by surgery and post-operative chemotherapy. In order to ameliorate survival rates of patients, new therapeutic approaches have been evaluated, mainly immunotherapy with antibody-drug conjugates or immunoconjugates. These molecules consist of a carrier (frequently an antibody) joined by a linker to a toxic moiety (drug, radionuclide, or toxin). Although several clinical trials with immunoconjugates have been conducted, mainly in hematological tumors, their potential as therapeutic agents is relatively under-explored in many types of cancer. In this review, we report the immunoconjugates directed against OS surface antigens, considering the in vitro and in vivo studies. To date, several attempts have been made in preclinical settings, reporting encouraging results and demonstrating the validity of the idea. The clinical experience with glembatumumab vedotin may provide new insights into the real efficacy of antibody-drug conjugates for OS therapy, possibly giving more information about patient selection. Moreover, new opportunities could arise from the ongoing clinical trials in OS patients with unconjugated antibodies that could represent future candidates as carrier moieties of immunoconjugates.

## 1. Introduction

Bone sarcomas are a group of rare malignant tumors that can arise from bone tissue, cartilage, and muscle, accounting for about 0.2% of all human neoplasms [1]. Among bone sarcomas, osteosarcoma (OS) is the most common primary bone malignancy. It is an aggressive osteoid-producing tumor of mesenchymal origin, characterized by a complex and, frequently, uncertain etiology. While most of the cases are sporadic, an increased risk for OS has been associated with radiation exposure, treatment with alkylating agents, and rapid bone proliferation. Predisposition to OS has been observed in association with Paget’s disease of the bone, Li-Fraumeni syndrome, hereditary retinoblastoma, Diamond Blackfan anemia, and autosomal recessive disorders like Rothmund-Thomson, Werner, Bloom, and RAPADILINO syndrome [2,3,4].

Typically occurring during the adolescent growth spurt, with a peak in the second decade of life and a higher incidence in males, OS shows a bimodal distribution with a second peak in the population over 65 years old, frequently associated with prior radiation therapy or Paget’s disease [5]. Although some OS are low-grade variants, the vast majority are high-grade malignancies with an elevated tendency for metastatic spread to the lungs. Survival rates for children and young adults at five years after diagnosis range from about 60 to 70%, for patients with non-metastatic disease, but only from 10 to 30% for those with metastatic disease at initial diagnosis [6]. Furthermore, even in the subset of patients with non-metastatic disease at diagnosis, about 40% of patients develop metastasis at later stages [7].

The current standard care for high-grade OS treatment is neoadjuvant chemotherapy, followed by surgery to achieve local control, and then post-operative adjuvant chemotherapy [8]. Several drugs, given in multiple chemotherapeutic regimens, have shown efficacy in OS: doxorubicin, cisplatin, high-dose methotrexate, ifosfamide, etoposide, and epirubicin, with the first three being the most frequently used [9,10,11,12]. Patients with relapsed or refractory disease are treated with different regimens, including radiotherapy with ^153^Samarium and ^223^Radium [13].

Patients with metastatic disease or recurrence, who suffer a worse prognosis, need new therapeutic approaches, in order to go beyond the above reported rates of survival.

## 2. Immunoconjugates for Cancer Therapy

Different drug delivery systems have been developed to maximize the therapeutic efficacy of anti-tumor compounds, reducing the effective dose and the systemic toxicity. The ideal way to satisfy these goals is to develop a drug whose activity is limited to malignant cells, having limited and tolerable side toxicity. Immunoconjugates represent an important tool towards this direction. These molecules consist of a carrier part, frequently a monoclonal antibody (mAb), whose function is to deliver a toxic moiety to specific tumor targets.

**Figure 1:**
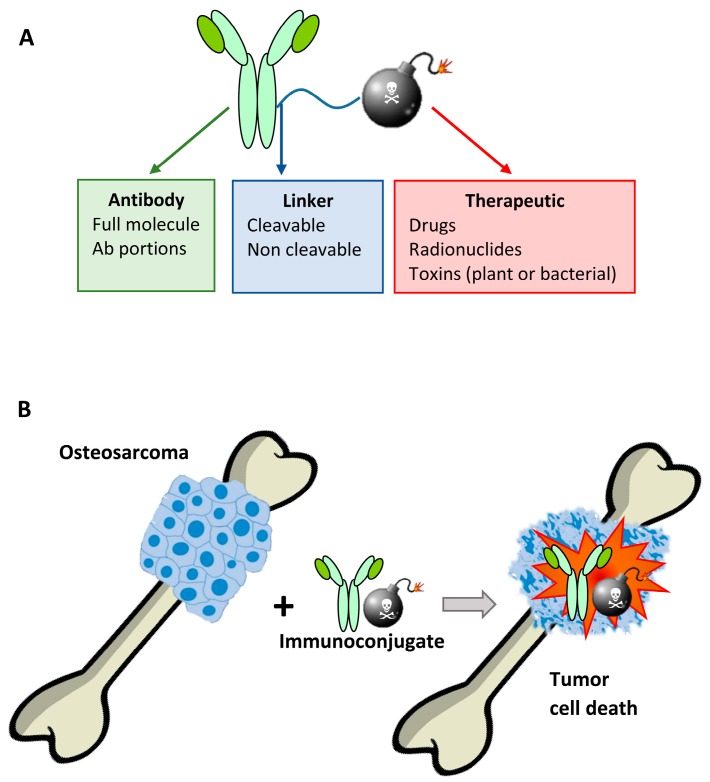
Scheme of a typical immunoconjugate structure (**A**) and antitumor activity (**B**).

The first immunoconjugates were produced using murine antibodies, which caused severe immunogenic reactions in patients developing anti-mouse antibodies. However, immunogenicity was subsequently reduced by the development of chimeric, humanized, and then fully human antibodies [14]. Three different classes of cytotoxic agents have been classically used to produce immunoconjugates: pharmacological agents, radionuclides, and toxins (both bacterial and plant) [15,16,17]. The carrier and the therapeutic moiety are mainly joined by a chemical linker [18] (Figure 1).

The linker can be cleavable or non-cleavable. The choice of a suitable linker is a crucial step in the design of an immunoconjugate, because it will affect not only the delivery of the payload to the target cell, thus determining its cytotoxic efficacy, but also the stability of the molecule and its pharmacokinetics. When the payload is a drug or a toxin, to obtain the maximal intratumoral delivery, the linker has to be highly stable in blood circulation, but capable of efficiently releasing the toxic moiety once it has arrived at the target. The strategies to select the best linker were recently reviewed by Beck and coworkers [19].

Amongst pharmacological agents, the most utilized are drugs that block tubulin polymerization, such as monomethyl auristatin E (MMAE) and maytansinoids (DM1, DM4), or that induce DNA strand breaks, such as calicheamicin. The main advantage of using drugs resides in a well-known toxicity profile and mechanism of action, whereas the main disadvantage derives from their action only on proliferating cells.

The most utilized radionuclides to obtain immunoconjugates are the radioactive isotopes of iodine and yttrium. Radionuclides exert their toxic effects not only on cells recognized by the carrier, but also on those located close to them. This fact represents an advantage because it can also eliminate tumor cells not expressing the antigen or expressing a mutated antigen, but it can be also a disadvantage, because of the unspecific toxicity for normal cells surrounding the tumor. Other disadvantages derive from the difficulty in handling radionuclides and in their rapid decay.

The third immunoconjugate class consists of “immunotoxins”, chimeric proteins in which an antibody is linked to a protein enzyme with cytotoxic activity, mainly of bacterial or plant origin. Pseudomonas exotoxin A and diphtheria toxin are the most used bacterial toxins to construct immunoconjugates specific for many different targets on cancer cells. Some of these conjugates have also been tested in clinical trials [20]. The most used plant toxins are the ribosome-inactivating proteins (RIPs), which are enzymes able to damage ribosomes and other substrates, in an irreversible manner, thus inducing cell death. RIPs are mainly divided into type 1, which consist of a single-chain protein such as saporin and pokeweed antiviral protein (PAP), and type 2, which are composed of an enzymatic A-chain linked to a B-chain possessing lectin properties, such as ricin [21,22]. Many clinical trials with RIP-containing immunoconjugates have been conducted and some are ongoing [23,24,25]. The use of toxins as pharmacologically active moieties of conjugates shows several advantages in comparison with drugs and radionuclides. Differently to drugs, toxins do not act in a stoichiometric ratio, but in a catalytic manner. Furthermore, they do not induce multidrug resistance (MDR) and are able to kill cells in both dividing and quiescent states. The main disadvantage of toxins, of both plant and bacterial origin, derives from their immunogenicity that after repeated administration leads to the production of blocking anti-toxin antibodies.

Despite several clinical trials with immunoconjugates having been conducted, mainly in hematological tumors, their potential as therapeutic agents is relatively under-explored in many types of cancer. At present, three antibody-drug and two antibody-radionuclide conjugates are currently approved by FDA for clinical use in cancer therapy. Mylotarg^®^ (gemtuzumab ozogamicin) is an anti-CD33 mAb linked to calicheamicin, approved from 2000 to 2010 for the treatment of acute myeloid leukemia; Adcetris^®^ (brentuximab vedotin, SGN-35) is an anti-CD30 mAb conjugated to MMAE, approved for relapsed/refractory Hodgkin lymphoma and anaplastic large-cell lymphoma; Kadcyla^®^ (trastuzumab emtansine) is an anti-HER2 mAb conjugated to mertansine (DM1), approved for HER2 positive breast cancer; and Zevalin^®^ and Bexxar^®^ are two anti-CD20 mAbs conjugated with ^90^Yttrium and ^111^Indium, respectively, approved for the treatment of Non-Hodgkin lymphoma. The availability of numerous antibodies targeting cancer-associated antigens represents an intriguing opportunity to utilize them as immunoconjugates, especially for pathologies needing new approaches, like OS.

## 3. Immunoconjugates for OS Experimental Therapy

Several immunoconjugates directed against certain OS surface targets have been explored in vitro and in vivo (Table 1). The encouraging results suggest the need to further explore the possibility to develop immunotargeting strategies for OS patients. Below, a list of the main immunoconjugates used to OS experimental therapy are reported, classified considering the molecular target.

### 3.1. Anti-gp72 mAb 79IT/36

The first attempts to develop an immunotherapeutic approach to OS cells were made using the anti-gp72 mAb 79IT/36. This antibody is not specific for OS cells, even if it preferentially binds tumor cells rather than normal tissue [26]. Initially, the results were not encouraging; in fact, when 79IT/36 was conjugated to the anti-mitotic drug vindensine, the resulting immunoconjugate was less effective toward OS cells than vindensine alone, suggesting a diminished drug activity after conjugation [27]. On the contrary, promising results were achieved using methotrexate and ricin toxin A chain (RTA) as therapeutic moieties. After conjugation of methotrexate to human serum albumin and 79IT/36 mAb, the drug efficiency on osteogenic sarcoma was significantly augmented [28]. Even better results were obtained with 79IT/36-RTA; this immunotoxin was found to be about two orders of magnitude more toxic to target cells than the 79IT/36-methotrexate conjugate. In vivo testing of the 79IT/36-RTA immunotoxin confirmed the ability of this immunoconjugate to inhibit tumor growth in mice. In five days, a total dose of immunotoxin of 25 mg/kg body weight produced a 75% inhibition of tumor growth. Prolonged treatments were found to be ineffective in ameliorating the outcome [26,29].

### 3.2. TP-3

TP-3 mAb recognizes an antigen expressed on the surface of OS cells, but showing a limited or null distribution on normal tissues [30]. TP-3 mAb was used to obtain immunoconjugates for OS experimental therapy. TP-3 conjugated with ^211^Astatine (^211^At) was tested on two OS human cell lines (OHS and KPDX) with different surface expression of the TP-3 ligand, with OHS cells being more highly expressed than KPDX. Consistently, the ^211^At-TP-3 reduced survival in both cell lines, but a higher cytotoxicity was reported for the OHS cells. ^211^At-TP-3 showed a low cytotoxic activity in a non-target melanoma cell line [31].

A different strategy to augment the immunoconjugate specific toxicity was attempted by the double conjugation of TP-3 to folate and radionuclides. The folate receptor is frequently expressed at high levels in several human cancers. This dual affinity immunoconjugate showed in vitro high binding property to OS cells. However, some doubts remain about its actual in vivo efficacy. In fact, in the same paper, a generic IgG did not change its biodistribution in mice after conjugation to folate, suggesting that the folate had no impact on in vivo distribution [32].

TP-3 was also used as a carrier moiety in the construction of immunotoxins, containing the RIP PAP or the bacterial toxin *Pseudomonas* exotoxin A (PE-A). TP-3–PAP specifically killed OS cells in vitro, showing a strong reduction of viability with an IC_50_ in the pM range, and eliminating more than 99% of OS cells in clonogenic tests. In vivo experiments in a mouse model of pulmonary metastases showed that the immunotoxin was able to reduce metastasis growth [33]. Pharmacokinetic and systemic toxicity of TP-3-PAP were investigated in BALB/C mice. Signs of toxicity were detected only at doses of 2 mg/kg and higher. The antitumor activity of TP-3-PAP was evaluated in vivo in SCID mice xenografted with OHS cells. When injected 2 h after tumor cell inoculation, the immunotoxin was able to delay tumor growth and progression. A cumulative dose treatment over three days of 0.25 and 0.50 mg/kg resulted in a mean tumor-free survival time of 26.5 and 32.0 days, respectively. Treatment of mice with 1.0 mg/kg resulted in 67 ± 19% of mice still alive and tumor-free at 150 days. In the same experiments, control mice survival was 19 days [34]. Recombinant immunotoxins were obtained using engineered versions of TP-3 and a truncated *Pseudomonas* exotoxin A. These recombinant immunotoxins maintained the ability to kill OS cells, but in vitro showed a 30-fold lower cytotoxicity with respect to TP3-PAP. In vivo administration of recombinant immunotoxins in SCID mice xenografted with OHS-M1 cells resulted in a complete regression of tumors [35,36].

### 3.3. Anti-gpNMB

Membrane glycoprotein NMB was demonstrated to be expressed in 92.5% of samples in a microarray analysis of OS from 67 patients. Intermediate to high staining was detected in 68.5% of samples, indicating that gpNMB could be a promising target for antibody-mediated drug delivery in OS [37]. Glembatumumab vedotin is a conjugate that combines the targeting properties of an anti-gpNMB mAb to the cytotoxic activity of the anti-mitotic agent MMAE. In vitro cytotoxic activity of glembatumumab vedotin was tested on several cell models, including 10 short-term cultures from patient derived OS cell lines, five human OS cell lines, and four human xenograft-derived cell lines maintained by serial passages in SCID mice. In 74% of the cell lines tested, the immunoconjugate demonstrated a cytotoxic effect with IC_50_ values ranging from 6 to 55 µg/mL. Cytotoxic activity was found to be significantly correlated to gpNMB protein levels in OS cells [37]. In vivo testing showed that glembatumumab vedotin possessed anti-tumor activity in pediatric preclinical models of OS xenografted in an SCID mouse. Glembatumumab vedotin was well tolerated with no dead animals and only minimal weight loss. The ratio of time to event for treated versus control animals was ≥2 in five out of six different OS xenograft models, with a complete response being maintained in three of them [38]. Glembatumumab vedotin has been evaluated in two phase I/II clinical trials on patients with advanced breast cancer and on patients with advanced melanoma. In both studies, this immunoconjugate had an acceptable and manageable safety profile [39,40].

Although several antigens were exploited for OS immunotherapy in preclinical studies, to date, only one clinical trial has been conducted with Glembatumumab vedotin (also known as CDX-011 or CR011-vcMMAE). This immunoconjugate is currently under clinical investigation in a phase II trial in adolescents and young adults with recurrent or refractory OS. This study started in February 2016 and is now ongoing, but no longer recruiting participants (NCT02487979).

### 3.4. Anti-Insulin-Like Growth Factor-2 Receptor

A significant overexpression of the cation-independent mannose-6-phosphate/insulin-like growth factor-2 receptor (IGF2R) was showed by Hassan et al., who analyzed cell surface receptors expression across several OS cell lines, suggesting the possibility to target this receptor for a therapeutic purpose [41]. The antitumor efficacy of a mAb anti-IGF2R linked to β-emitter ^188^Rhenium on IGF2R-expressing OS cell lines was tested in vivo in SCID mice xenograft models. A marked suppression of tumor growth was demonstrated in mice treated with the radioimmunoconjugate at day 24 after treatment, suggesting a potential therapeutic efficacy in OS patients with IGF2R-overexpressing tumors [42].

### 3.5. Other Surface Markers

Several other surface markers have been tested in preclinical settings as potential targets for antibody-mediated drug delivery in OS treatment. CD146 is a tumor-associated cell surface glycoprotein overexpressed in several cancers, including OS. Radiolabeled anti-CD146 OI-3 mAb conjugated with ^125^Iodine or ^177^Lutetium showed promising results in nude mice in terms of tumor uptake and in vivo stability, but further investigations are needed to assess the anti-tumor efficacy of these radioimmunoconjugates [43].

CD166 (activated leukocyte cell adhesion molecule, ALCAM) was targeted in vitro with anti-CD166 mAb conjugated to doxorubicin-loaded liposomal nanoparticles. Antibody-targeted liposomal nanoparticles compared to non-targeted liposomal nanoparticles showed an increase in cytotoxicity for OS cells, ranging from two- to 23-fold, depending on liposomal formulation and the cell line tested [44].

CD184 (CXCR4) is a G-protein coupled receptor that has been found to be highly expressed on the surface of metastatic tumor cells and to be efficiently internalized after ligand binding. A recombinant anti-CD184 mAb conjugated to MMAE showed a high in vitro toxicity on metastatic OS cells obtained by lung metastasis formed by a human OS cell line implanted in a mouse tibia. In a tumor xenograft lung-seeding model, tumors were not detectable at 35 days in mice receiving the immunoconjugate i.v. at 3 doses/2.5 mg/kg once every five days [45].

CD248 (endosialin/tumor endothelial marker 1) is a surface protein overexpressed in about 50% of sarcomas. A fully human anti-CD248 mAb conjugated to MMAE showed growth inhibition efficacy in vitro, in CD248 overexpressing OS cells, with IC_50_ of 1.5 µg/mL mAb at 96 h of treatment [46].

## 4. Future Perspectives and Conclusions

Considering that in the last 30 years currently available therapeutic options for OS treatment failed to improve patients’ outcome, there is a need for a new therapeutic strategy, especially for patients with metastatic disease at diagnosis, who have a poor prognosis. The investigation of the ideal antigen for OS immunotherapy is still one of the main goals for researchers involved in this field, because of the high genetic instability and pleomorphism of OS cells. To date, several preclinical studies have reported encouraging results, thus demonstrating the validity of the idea. Moreover, the clinical experience with glembatumumab vedotin provides new insights into the real efficacy of antibody-drug conjugates for OS therapy in a clinical setting, possibly giving more information about patient selection.

To date, the main limitation in the clinical use of immunoconjugates has been their low therapeutic index. An important limitation in the use of immunoconjugates derives from their cross-reactivity with normal tissues, because target antigens are rarely restricted to cancer cells. Moreover, the conjugation process can alter the pharmacokinetic and biodistribution of both components, varying their toxicity for vital organs, such as the bone marrow, kidney, and liver, etc. In the case of drugs containing immunoconjugates, limitations also derive from the instability of the conjugate and drug release in the bloodstream, at least with first generation linkers, and from the selection of drug resistant cells. However, in the last years, new generations of immunoconjugates have been obtained optimizing the linker and the conjugation chemistry [19], giving good future perspectives in the field of drug containing immunoconjugates. The MDR problem can be overwhelmed using toxins or radionuclides for which MDR has never been described. Moreover, in silico biology can represent a valuable tool for the identification of new potential targets in oncology research. In 2012, Orentas and coworkers identified a list of potential surface tumor antigens to be targeted by antibody therapies using an in silico approach based on the analysis of expression databases for pediatric tumors. Their algorithm identified 31 hits in OS samples, corresponding to tumor-associated antigens that may be potentially exploited for immunotherapy [47]. However, to date, none of these potential surface targets have been investigated in preclinical or clinical studies for OS therapy.

Moreover, human OS specimens show a high expression of the endocytic collagen receptor, uPARAP/Endo180, which can be exploited for specific tumor targeting [48]. As uPARAP/Endo180 is a rapidly recycling endocytic receptor, it represents an ideal candidate for immunotargeted drug delivery. An anti-uPARAP/Endo180 mAb was conjugated to MMAE. The resulting immunoconjugate was tested in vitro on several sarcoma cell lines, but not OS, showing encouraging results. Thus, this conjugate could also find useful applications in OS that highly express this antigen [49].

A different exploited strategy to attack cancer is the targeting of its blood vessels. This approach can be particularly valid in sarcoma because of their rich neovasculature. The endoglin targeting with immunoconjugates is an attractive approach to actively suppress the blood supply to the tumor [50].

It has been proven that the antitumor effect of antibodies or immunoconjugates can be strongly augmented when they are administrated in combination with other antineoplastic drugs [51,52]. These combined therapies represent a future perspective in OS treatment with immunoconjugates.

Furthermore, it should be considered that some clinical trials with unconjugated antibodies are currently ongoing for recurrent OS immunotherapy: -NCT02502786, Humanized mAb 3F8 (anti-glycolipid GD2) in combination with GM-CSF for the treatment of recurrent osteosarcoma (Phase II); -NCT02484443, Dinutuximab (anti-glycolipid GD2) in combination with sargramostim for treating patients with recurrent OS (Phase II); and -NCT01614795, Cixutumumab (anti-human insulin-like growth factor-1 receptor), and Temsirolimus for treating younger patients with recurrent or refractory sarcoma (Phase II). New opportunities could also arise from these trials in terms of carrier availability to conjugate with toxic molecules.

## Figures and Tables

**Table 1:** Immunoconjugates tested for OS therapy.

Antibody	Target	Cytotoxic Agent	In Vitro	In Vivo	Clinical Trial	Ref.
79IT/36	gp72	vindensine	√	-	-	[27]
		methotrexate	√	-	-	[28]
		ricin A chain	√	√	-	[26,29]
TP-3	80 kDa sarcoma associated antigen	^211^At	√	-	-	[31]
		PAP	√	√	-	[33,34]
		PE-A	√	√	-	[35,36]
Glembatumumab	gpNMB	MMAE	√	√	√	[37,38]
MEM-238, MOPC21	IGF2R	^188^Re	√	√	-	[42]
OI-3	CD146	^125^I, ^177^Lu	√	√	-	[43]
anti-ALCAM cys-diabody	CD166	doxorubicin	√	-	-	[44]
A122pAcF	CD184	MMAE	√	√	-	[45]
fully-human anti-endosialin Ab	CD248	MMAE	√	√	-	[46]

The symbol √ means that the immunoconjugate has been tested, while the symbol - means that the immunoconjugate has not been tested in vitro, in vivo or in clinical Trial.
